# Instrumental Music in the Asylum of Quatre-Mares

**Published:** 1860-01-01

**Authors:** Legrand du Saulle


### Art. VI.—INSTRUMENTAL MUSIC IN THE ASYLUM OF QUATRE-MARES.

BY DR. LEGRAND DU SAULLE.

If we recollect rightly it is more than twenty years since Drs. Parchappe and Debouteville started a class of vocal music in the important asylum of St. Yon, and gave there a series of private concerts, and even an annual public *fête,* in which the chorus took the principal part. This tradition is preserved at St. Yon, and the establishment at Quatre-Mares, which was opened for patients on the 1st January, 1852, has soon followed the example of its senior. Some difficulties, however, presented themselves upon the separation of the men from the women. Nothing, in fact, is more difficult than to make lunatics of the same sex sing in different tones, whatever may be their voice, and nothing becomes more tiresome than a psalm-tune sung by forty or fifty voices in unison. In Germany these things come about differently. There the appreciation of varied intonation is developed naturally and without instruction; the least well-to-do classes of the population possess this musical taste, which with us, in most cases, is only obtained as the result of care and study. In all probability many years will elapse before our Orpheists will have been able to propagate this taste, which we are, perhaps, too much in the habit of considering as an aptitude inherent in certain nationalities.

However this may be, Dr. Dumesnil, chief physician of the asylum at Quatre-Mares, soon ascertained that all the efforts of a professor would have little or no success in any attempt to teach part singing methodically to a number of lunatics.

But, seconded by a very zealous superintendent, M. Goubaux, and by a very distinguished professor of instrumental music in Rouen, he saw a possibility of teaching the patients under treatment to perform some pieces together upon wind instruments. Several of the attendants also gave their assistance to the work, but these two elements taken, so to speak, from the most mobile portion of the inhabitants, were not sufficient to form a proper nucleus. It was then attempted to incorporate in the band patients considered to be incurable, and the sojourn of whom in the asylums of the Seine-Inferieur sometimes continues for twelve or fifteen years.

The success obtained by M. Dumesnil is truly surprising, and we have been told that the inspector-general, M. Parchappe, at his last visitation of the asylum, exhibited very marked satisfaction, and even a certain emotion, on seeing poor invalids whom he had long left in an intellectual condition not susceptible of cure, answer correctly numerous questions on the principles of musical art, and play with precision and considerable taste, a series of pieces which would do honour to more than one society of amateurs. We can speak of this from our own knowledge, for during a recent excursion we made in Normandy, we received, on the strength of our having been a former medical resident in the asylum, a serenade at our arrival and departure. We were fully sensible of this unexpected honour, and we felt the greatest pleasure in listening to a series of selections from, and overtures of, standard operas. For about two years, fifty or sixty lunatics have taken part in these exercises. There are never less than fifteen patients there, and it is seldom that a convalescent leaves the asylum, however little disposed towards music, without having been a member of the musical corps.

This useful and powerful distraction gives the performers an unwonted expression of countenance; the features of the demented and melancholic become animated, and assume an air of gaiety; even the maniac under a certain degree of excitement, follows his notes with regularity, and beats measure with an imperturbable manner, though marking his stops perhaps by reflections, signs, and gestures, which are not all on the programme! Dr. Dumesnil is convinced, together with his colleague, Dr. Viret, that the hallucinated patients, and even those who are only present as spectators, escape from their delirious conceptions during these performances. This favourite influence is not less marked over the minds of the other invalids whether they are confined to their several departments, or whether with drums and music at the front, they take long walks in the domain of the establishment, or in the neighbouring woods.

These remarkable results have so struck the Administrative Board, that every demand to facilitate and propagate musical taste amongst the insane has been warmly welcomed by the Commission of Inspection, and immediately approved by the Prefect of the Seine-Inferieur. We know for a fact that a single purchase of brass instruments alone has been made to the amount of 1500 francs. But in no direction does the superior administration exhibit more benevolent care, greater sacrifices, and more sympathizing interest than in the department relating to the treatment of the insane. We also congratulate M. Dumesnil in that he never has occasion to present any request for the well-being of the unhappy sufferers, to whom he consecrates with such success the remarkable administrative capacity, the consummate medical tact, and the prodigious activity with which he is endowed, without such request being at once understood and granted.

May the *harmony* of the Asylum of Quatre-Mares have more than one echo in our other lunatic establishments. Some are said to stand in much need of it.

(*Annales Medico-Psychologiques.*)

